# Quantifying uncertainty due to sampling error in a social vulnerability index: A Monte Carlo approach

**DOI:** 10.1371/journal.pone.0354333

**Published:** 2026-08-03

**Authors:** Jawata A. Saba, Kevin D. Ash

**Affiliations:** Department of Geography, University of Florida, GainesvilleFlorida, United States of America; Bihar Agricultural University, INDIA

## Abstract

Social vulnerability indices are widely used to assess communities’ relative vulnerability to environmental hazards. However, existing social vulnerability indices, including the CDC’s Social Vulnerability Index (CDC SVI), rely on deterministic scores that do not account for uncertainty from sampling error in American Community Survey (ACS) data. This study introduces SVI-MC, a probabilistic approach that leverages 10,000 Monte Carlo simulations to quantify aleatoric uncertainty in the SVI. Our method embeds sampling variability directly into index calculations, producing uncertainty aware composite vulnerability estimates. Results from eight southeastern U.S. states using census tract level data reveal that Theme 2 (Household Characteristics) exhibits the highest uncertainty, while Theme 3 (Race & Ethnicity) is the most stable. Rural, mountainous, and coastal areas display greater classification instability, while urban tracts are generally more stable. Additionally, we introduce an entropy-based measure that complements coefficients of variation (CVs) by capturing instability in categorical rankings. Comparisons with the CDC SVI show that while 68% of tracts align in the most and least vulnerable categories, one-third experience ranking shifts in our model. SVI-MC provides a more comprehensive and uncertainty-aware approach to social vulnerability assessment. This method is scalable, replicable, and adaptable, offering future applications for larger study regions or at finer observation units. The findings underscore the need for uncertainty-aware mapping in disaster planning, emergency response, and public health research, supporting more informed decision-making.

## Introduction

Vulnerability broadly refers to the potential for loss or harm in relation to environmental hazards [1–3]. In research on hazards, disasters, and climate change, vulnerability is commonly understood as a function of exposure, sensitivity or susceptibility, and adaptive capacity [2,4–6]. Exposure refers to the extent to which people, assets, or systems are subject to harm, while sensitivity and adaptive capacity describe the social, economic, institutional, and material conditions that shape the ability of people and places to anticipate, withstand, respond to, and recover from adverse impacts [4,6–8].

Social vulnerability focuses specifically on the social and economic conditions that make some populations and places more susceptible to harm, even when exposed to similar hazards. These conditions include factors such as socioeconomic status, gender, race/ethnicity, age, employment, housing tenure and quality, family structures, education, access to transportation and medical services, and other indicators of social dependence or resource access [7,8]. Because social vulnerability is complex and not directly observable, it is often operationalized through composite indices that combine multiple demographic and socioeconomic indicators to identify places where vulnerability-related characteristics overlap.

Social vulnerability indices have been prevalent in hazards geography and interdisciplinary disasters research for over twenty years. These indices can be useful to understand the vulnerability of people and places, to prepare for and mitigate impacts for future hazards, and to study recovery from past hazard events. However, the socioeconomic data underpinning these models are used often without sufficient attention to uncertainty from survey sampling error. Many commonly used indicators are derived from the US Census Bureau’s American Community Survey (ACS), meaning they are survey estimates rather than exact counts or percentages. Although recent work has emphasized that socioeconomic vulnerability assessments involve multiple forms and sources of uncertainty, uncertainty in social vulnerability research remains less systematically developed than in many areas of physical climate and hazard science, including meteorological, hydrological, and climate modeling [9]. Recognizing this gap, a growing body of literature has begun to examine the robustness, sensitivity, and uncertainty of social vulnerability indices [10–13].

## Background

### Social vulnerability index

Social vulnerability is a complex phenomenon with no single, universally accepted method for measuring it. The concept has been applied across multiple disciplines, including urban planning, the social sciences, geography, and ecology, each with distinct perspectives and measurement approaches [14,15]. In hazards and disaster research, it is commonly operationalized through composite indices that combine multiple socioeconomic and demographic indicators into a single score or ranking [17]. These indices are useful heuristic tools for identifying places where vulnerability-related characteristics overlap, but their construction requires several methodological decisions, including selection, scaling, weighting, aggregation, and validation against observed hazard impacts [10,11,16].

Among the quantitative tools used to assess social vulnerability, two indices have emerged as the most prominent in research and applied practice: the Social Vulnerability Index (SoVI) and the CDC Social Vulnerability Index (CDC SVI). SoVI was developed by the Hazard, Vulnerability & Resilience Institute (HVRI) at the University of South Carolina and uses an inductive approach in which multiple variables are reduced using principal component analysis and summed to create an index score [17–22]. Recent versions of SoVI use ACS 5-year estimates for nearly all data inputs [23].

The CDC SVI is a hierarchical index in which 16 variables are grouped into four themes: Socioeconomic Status, Household Characteristics, Racial and Ethnic Minority Status, and Housing Type and Transportation [24]. For each indicator, census tracts or counties are normalized using percentile ranks. These percentile ranks are summed within each theme to create subindices, and the theme-level percentile ranks are then summed to produce the final composite SVI score [24]. The final output ranges from 0 to 1, with higher values indicating greater relative vulnerability.

For this study, we focus on the CDC SVI for three reasons. First, previous studies suggest that hierarchical vulnerability indices such as the CDC SVI may be more robust in index construction than inductive approaches [10,11]. Second, the CDC SVI is relatively straightforward to replicate, making it well suited for an extension from a deterministic index to a probabilistic framework. Third, the CDC SVI is widely used in hazard, emergency management, and public health applications, including FEMA’s National Risk Index (NRI) [25]. Because the CDC SVI relies on ACS estimates and percentile rankings, uncertainty in the input data can affect not only the final index values but also the relative rankings and mapped classifications of census tracts. Therefore, this study develops a probabilistic version of the CDC SVI that uses ACS margins of error to quantify uncertainty in the four theme-level subindices and the final composite index.

### Uncertainty in ACS data and vulnerability indices

The CDC SVI relies on American Community Survey (ACS) 5-year estimates because these data are available for all counties and census tracts in the US. Unlike decennial census counts, ACS values are survey estimates and are therefore published with margins of error (MoE) that quantify sampling uncertainty. This uncertainty is especially relevant at smaller geographic units, such as census tracts, where sample sizes are smaller and MoEs can be relatively large [26–29].

The ACS calculates sampling error using the Successive Difference Replication (SDR) method [30].In this method, 80 replicate estimates are generated from the original survey sample. The variance is estimated by summing the squared differences between each replicate estimate and the full-sample estimate and multiplying this sum by 4/80. The standard error is the square root of this variance, and the MoE is calculated as 1.645 times the standard error, corresponding to a 90 percent confidence level (Equations 1–3) [31]. These MoEs provide the basis for quantifying how sampling uncertainty in ACS estimates propagates through the SVI.

Equation 1: Successive Difference Replicate Formula


$$\begin{array}{c} Variance=\frac{\text{4}}{\text{8}\text{0}}\sum_{i=\text{1}}^{\text{8}\text{0}}{\left({Var\_Rep}_{i}-\text{Estimate}\right)}^{\text{2}} \end{array}$$

Equation 2: Standard error


$$\begin{array}{c} Standard\;Error=\sqrt{\text{Variance}} \end{array}$$

Equation 3: Margin of Error


$$\begin{array}{c} Margin\;of\;Error\;(MoE)=\text{1}.\text{6}\text{4}\text{5}*Standard\;Error \end{array}$$

Two types of uncertainty are especially relevant for vulnerability modeling. Epistemic uncertainty arises from modeling choices, such as indicator selection, scaling, weighting, and aggregation [11,32]. Aleatoric uncertainty arises from inherent variability in the data-generating process, including sampling error in survey estimates [11]. This study focuses on aleatoric uncertainty because ACS MoEs formally quantify sampling uncertainty in the input data used to construct the CDC SVI. Prior research has examined uncertainty and robustness in vulnerability indices, including sensitivity to scale, geographic extent, and other model construction choices [10–13,33]. However, ACS sampling uncertainty is still often ignored in deterministic vulnerability maps and index scores, even though related work has shown that ACS uncertainty can affect spatial analysis, mapping, and composite indicators [27,28,34–38].

Recent hazards’ geography research has begun to address this issue more directly. Arabadjis et al. propagated uncertainty through a simplified wildfire risk index combining one ACS-derived social vulnerability indicator, the proportion of the population aged 65 and older, with a remotely sensed locational vulnerability indicator used as a proxy for live fuel moisture [39]. Their study demonstrates that uncertainty in input data can destabilize conclusions and map interpretations, potentially affecting resource allocation for prevention, mitigation, and recovery. This work is closely related to our study because it foregrounds uncertainty propagation in vulnerability and risk index construction. However, their social vulnerability component is a single ACS-derived variable, whereas our study propagates ACS sampling uncertainty through the full CDC SVI framework, including 16 indicators, four themes, percentile rankings, and the final composite index.

Most directly related to our study, Feldmeyer et al. (2026) proposed a framework to address the ACS data uncertainty in vulnerability modeling using variance replicate estimates (VREs) and Monte Carlo simulation [40]. Their work demonstrates that sampling error can propagate through composite index construction and affect index scores across alternative normalization, weighting, and aggregation choices. This reinforces the importance of treating sampling uncertainty as a core issue in composite indicators rather than as a minor data-quality concern. Building on this emerging direction, our study focuses specifically on the CDC SVI and examines how ACS sampling uncertainty propagates through it percentile-rank structure, theme-level subindices, final composite rankings, and mapped classifications. Together, this emerging literature highlights the importance of uncertainty propagation while also underscoring the need for index-specific applications that show how uncertainty affects widely used operational tools such as the CDC SVI.

Beyond the hazards and social vulnerability literature, researchers have also examined analytical problems associated with uncertainty in ACS estimates. Napierala and Denton investigated how ACS margins of error affect residential segregation measures for core-based statistical areas using dissimilarity indices [41]. Jung et al. compared spatial autocorrelation patterns of teen birth rates in Mecklenburg County, North Carolina, using both decennial census data and ACS estimates, finding meaningful differences in spatial autocorrelation statistics and emphasizing caution when interpreting spatial patterns derived from uncertain ACS estimates [42]. Donegan and colleagues studied spatial inequalities in health and mortality in relation to household income using the Index of Concentration at the Extremes (ICE) at the county level across the United States [43]. Using Census Bureau Variance Replicate Estimates (VREs), they found considerable uncertainty in county quintile membership due to ACS sampling error [43]. Similarly, Boscoe and coauthors estimated uncertainty in a Yost Index, a multivariate socioeconomic index used in cancer studies, at the census tract level in the Savannah, Georgia, area [44]. Their study also used Census Bureau VREs to compute margins of error for their composite index [44].

This study is guided by two main research questions. First, how can we quantify uncertainty in a social vulnerability index that results from sampling error in the underlying ACS data? Second, how does quantification of uncertainty in a social vulnerability index influence interpretation and visualization of the index values? This study will test the following hypotheses:

- H1: Because people living on lower incomes are less likely to participate in the ACS [44], the most uncertain and unstable input of the social vulnerability index will be Theme 1, which is labeled as Socioeconomic Status.
- H2: Uncertainty in social vulnerability index values as measured using information entropy will add unique information alongside a common uncertainty measure such as coefficient of variation because the entropy measure will capture *interactions* of uncertainty between census tracts when they are ranked for the purpose of constructing the index and for mapping of results.
- H3: Social vulnerability index values for census tracts in rural areas will be more uncertain and, therefore, more unstable in their categorization as among the most or least vulnerable, in comparison to social vulnerability index values for census tracts in urban areas.

## Data

### Study area and unit of analysis

**Fig 1:**
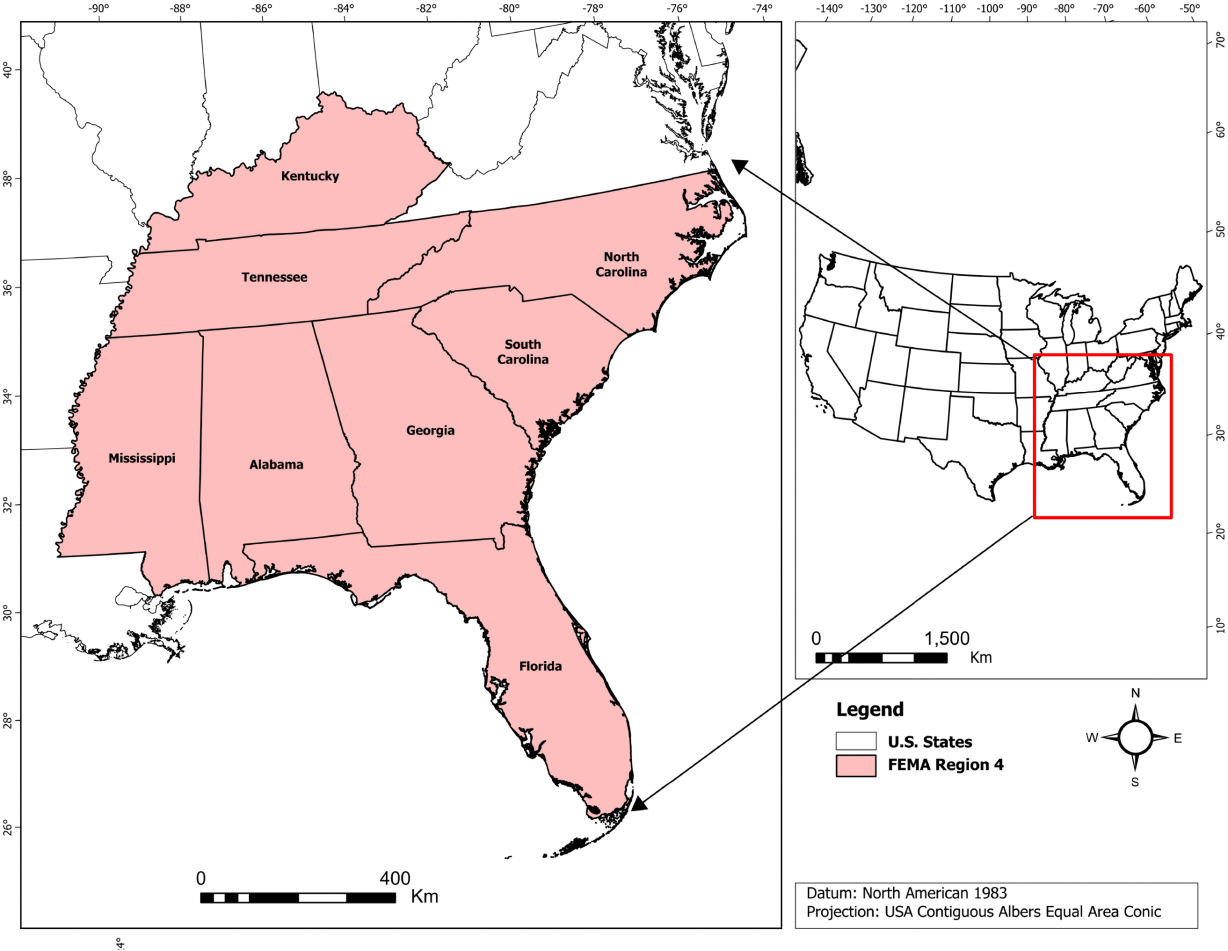
Study area.

The study area for this analysis is FEMA Region 4, and the geographic units of observation are census tracts. FEMA Region 4 is in the southeastern US (Fig 1) and is subject to a wide range of high-impact natural hazards, coinciding with a relatively dense population. The study area includes eight states: Florida, Alabama, Georgia, South Carolina, North Carolina, Tennessee, Mississippi, and Kentucky (FEMA, accessed in March 2024). We chose to conduct the analysis at the census tract level because the uncertainty in ACS data, while still somewhat problematic at the county level, becomes more relevant and problematic for analysis and decision-making at smaller sample sizes, such as can occur in census tracts [27,34]. The region has 17,208 total census tracts. In the analysis we used 17,113 census tracts because 95 of the census tracts have a zero-population count. All the shapefiles used in this study have been downloaded from the NHGIS website [45].

The study area of this research is FEMA region 4, which includes eight states: Florida, Alabama, Georgia, South Carolina, North Carolina, Tennessee, Mississippi, and Kentucky. Geographic boundary files are from IPUMS NHGIS, University of Minnesota. NHGIS citation and use terms were followed. No proprietary basemap tiles, satellite imagery, aerial imagery, or previously published copyrighted map images were used.

### Social vulnerability data

The CDC provides easy and public access to county and tract level SVI data on the SVI website [46], and the dataset also contains MoE for the 16 input indicators, as originally determined for the ACS 5-year estimates by the Census Bureau (Table 1). Individuals can download the raw estimates and MoE of the 16 indicators from the ACS website [47] and create their own SVI using CDC/ATSDR methodology [24]. Since SVI is already publicly available, we collected the SVI data at the census tract level from the CDC/ATSDR website to save time [48]. All data used in and generated from this research are made publicly available.

**Table 1:** Variables and their source table in ACS 5-year estimates from 2018-2022. Table adapted from SVI 2022 documentation. Source: [48,49].

Themes	Variables	ACS 2022 table
Socioeconomic Status (Theme 1)	Persons below 150% poverty	S1701
	Civilian (age 16+) unemployed	DP03
	Housing cost-burdened occupied housing units with annual income less than $75,000	S2503
	Persons (age 25+) with no high school diploma	B06009
	Uninsured in the total civilian noninstitutionalized population	S2701
Household Characteristics (Theme 2)	Persons aged 65 and older	S0101
	Persons aged 17 and younger	B09001
	Civilian noninstitutionalized population with a disability	DP02
	Single-parent household with children under 18	B11012
	Persons (age 5+) who speak English “less than well”	B16005
Racial & Ethnic Minority Status (Theme 3)	Minority (Hispanic or Latino (of any race); Black and African American, Not Hispanic or Latino; American Indian and Alaska Native, Not Hispanic or Latino; Asian, Not Hispanic or Latino; Native Hawaiian and Other Pacific Islander, Not Hispanic or Latino; Two or More Races, Not Hispanic or Latino; Other Races, Not Hispanic or Latino)	DP05
Housing Type & Transportation (Theme 4)	Housing in structures with 10 or more units	DP04
	Mobile homes	DP04
	At household level (occupied housing units), more people than rooms	DP04
	Households with no vehicle available	DP04
	Persons in group quarters	B26001

**Fig 2:**
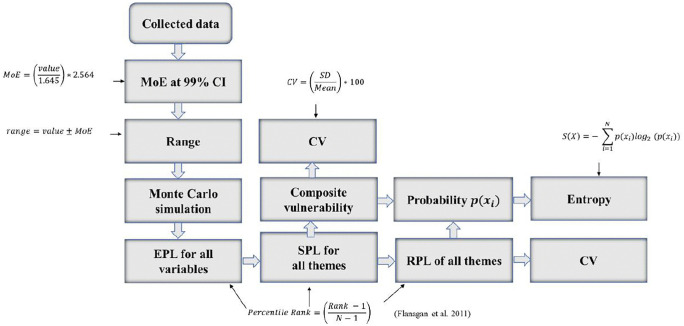
Methodology flowchart.

The SVI is organized as a composite index consisting of four themes: Socioeconomic Status, Household Characteristics, Racial and Ethnic Minority Status, and Housing Type and Transportation (Fig 2). Table 1 lists the 16 indicators that constitute these themes, along with their ACS source tables [24]. Briefly, Theme 1 captures socioeconomic disadvantage; Theme 2 captures age, disability, household composition, and language-related characteristics; Theme 3 captures racial and ethnic minority status; and Theme 4 captures housing type, crowding, group quarters, and transportation access. We use these four themes and their associated indicators as the basis for propagating ACS sampling uncertainty through the SVI.

## Methodology

We developed a novel approach to quantify and map the uncertainty inherent in the SVI stemming from the sampling error in the ACS input data. To address the first research question, we organized the methodology into discrete stages (Fig 2). Initially, we delineated upper and lower error bounds for each variable and census tract (hereafter referred to as tracts). First, we calculated the upper and lower bounds for the SVI percentage variables, which are indicated by the prefix EP using the CDC’s terminology. Given that the MoE provided in the ACS data is associated with a 90% confidence interval (CI), indicating a 10% probability of the actual estimate lying beyond this range, we recalibrated the MoE to establish a 99% CI, thereby generating new upper and lower bounds. This was done by converting the MoE values back to standard errors by dividing by 1.645, then we multiplied by 2.576 to derive the 99% CI. Although the 90% CI is conventionally utilized for uncertainty in ACS data, elevating the CI to 99% allows for a more comprehensive probabilistic examination of potential variations in the percentile ranks.

Subsequently, we calculated the upper and lower bounds for each percentage variable by adding and subtracting the recalibrated 99% MoE from each ACS point estimate. These bounds were used to parameterize a normal distribution for each variable in each census tract. Specifically, the ACS point estimate was treated as the mean of the distribution, and the standard deviation was derived from the 99% confidence interval. Utilizing a Monte Carlo approach, we then generated 10,000 simulated values for each variable by drawing from this normal distribution. After simulation, values were post-processed to ensure valid ranges before calculating percentile ranks; for example, percentage variables were constrained to the feasible range of 0–100. This approach is consistent with the ACS reporting of margins of error as functions of standard errors and confidence intervals. For each simulation, we recalculated percentile ranks for the 16 SVI indicators, summed the ranked indicators within each theme, calculated theme-level percentile ranks, and then summed the four theme ranks to produce the final simulated SVI value. Finally, we calculated coefficients of variation (CVs) across the 10,000 simulations to assess uncertainty in the theme-level and composite SVI values (Fig 2). Upon completing these calculations, we joined the output values to their corresponding census tracts in ArcGIS Pro to facilitate visualization of the data through mapping.

To address the second research question, we used the results from the Monte Carlo simulations to demonstrate how SVI values varied among census tracts given the uncertainty in the ACS data [11,16]. We compared the CDC’s 2022 SVI with the mean values derived from the 10,000 simulations of the index (SVI-MC) to evaluate shifts in percentile ranks. We also employed information entropy to quantify the instability or uncertainty in the SVI and its four sub-indices. Information entropy measures the degree of variation in ranked values when uncertainty in input data causes changes in the ordering of observational units [50,51]. In a geospatial context, Shannon entropy (equation 4) can be used to assess the uncertainty associated with assigning geographic units (tracts) to different classes in a choropleth map [52,53]. Lower entropy values indicate more stable classifications, while higher values signify greater uncertainty and less stability.

Equation 4: Information entropy


$$\begin{array}{c} \text{S}(\text{X})=-\sum_{i=\text{1}}^{n}{p(x}_{i}){log}_{\text{2}}(p\left(x_{i}\right)) \end{array}$$

We used RStudio to conduct the Monte Carlo simulations and associated statistical analyses and ArcGIS for all mapping and geospatial analyses. The mapping process involved joining the tract-level results from the SVI-MC simulations with the corresponding census tract shapefiles provided by National Historical GIS (NHGIS) [45]. All maps were produced in the Albers Equal Area Conic projection, which preserves area and is appropriate for regional-scale analyses in the United States. For choropleth visualization of the probabilistic SVI results, we applied a quantile classification scheme (five classes, or quintiles) [54] consistent with the CDC/ATSDR SVI methodology. Additional uncertainty surfaces were mapped using the same classification scheme for comparability. Spatial clustering and autocorrelation were evaluated using Moran’s I and the Getis-Ord Gi* hotspot statistics to identify areas where uncertainty and vulnerability values were significantly clustered.

We then assessed the frequency of rank changes for individual tracts across all 10,000 simulations to calculate the uncertainty of classification based on ranked order. In this study, entropy values closer to zero indicate that the SVI-MC and its four sub-index values are more stable across simulations, whereas values closer to one indicate greater instability due to larger ACS margins of error and rank shuffling among census tracts. We applied entropy to the quantile classification (five categories) and to the 90^th^-percentile flag used by the CDC to distinguish tracts with the highest levels of social vulnerability [24]. This approach allowed us to determine how frequently each tract occupied different quintiles across simulations. Finally, we analyzed whether tracts with higher entropy exhibited spatial clustering and assessed associations between tracts with elevated uncertainty, measured using coefficient of variation (CV), and those characterized by lower socioeconomic status or rural population density [44].

## Results

**Fig 3:**
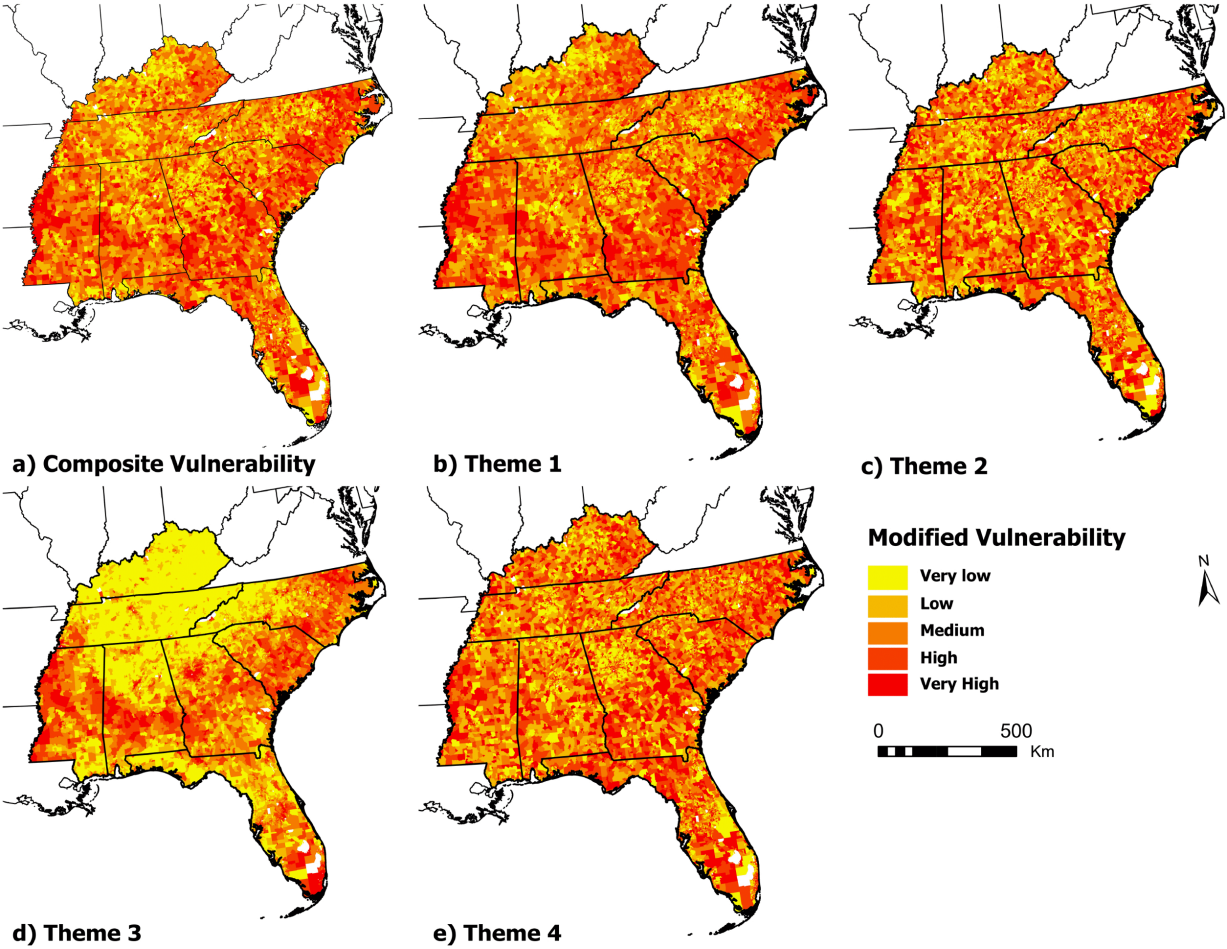
SVI-MC. The mean values from the Monte Carlo simulations of the a) SVI-MC composite index and four vulnerability themes or sub-indices: b) socioeconomic status; c) household characteristics; d) racial and ethnic minority status; and e) housing type and transportation. Geographic boundary files are from IPUMS NHGIS, University of Minnesota. NHGIS citation and use terms were followed. No proprietary basemap tiles, satellite imagery, aerial imagery, or previously published copyrighted map images were used. The mapped classifications and probabilities are author-generated outputs from the SVI-MC analysis.

**Fig 4:**
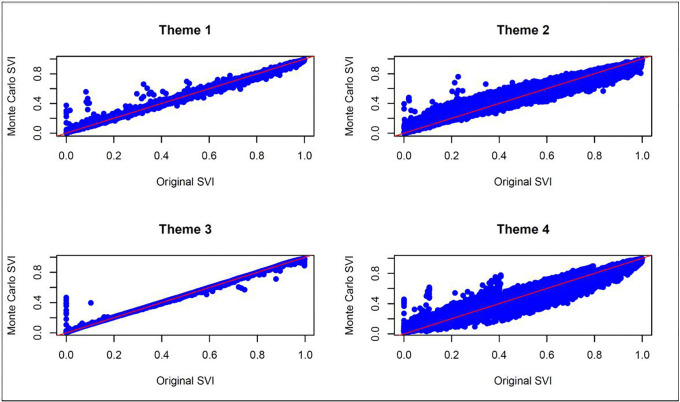
Scatter plots between the CDC SVI from 2022 and SVI-MC.

In this section, we present several results from the MC simulations using the CDC SVI data from 2022. First, we calculated the mean value for each census tract of the overall composite SVI measures across the 10,000 MC simulations, which we refer to here as SVI-MC (Fig 3). Then, as a quality check, we analyzed the deterministic census tract level CDC SVI values from 2022 with the SVI-MC mean values to check whether our code and resulting index simulations were consistent with the underlying CDC SVI, assuming that the mean SVI from the MC simulations would be highly correlated with the CDC SVI. We calculated the Pearson correlation between the CDC SVI and the mean of the SVI-MC composite vulnerability models, yielding a value of 0.99. This indicates a strong positive correlation between the two models. Looking further at the correlations between the four themes within SVI, the scatter plots (Fig 4) illustrate that more variability exists for Theme 2 (Household Characteristics) and Theme 4 (Housing Type and Transportation). In contrast, Theme 3, which has only one aggregated race/ethnicity variable, shows the least variation between the deterministic SVI data and the probabilistic SVI model (Fig 4).

We assessed the reliability of the SVI composite index and each theme sub-index using Esri’s guidance for the Coefficient of Variation (CV) (Equation 5). According to Esri [55], data is considered highly reliable if the CV falls between 0 and 12. A CV between 12 and 40 indicates moderate reliability, while a CV greater than 40 suggests low reliability. Based on these thresholds, we also categorized tract-level CV values from our SVI-MC simulations into three levels: low, medium, and high reliability, and mapped them for both the composite vulnerability and the four themes in our study.

Equation 5: We calculated the CV using this formula


$$\begin{array}{c} CV=\left(\frac{Standard\;Deviation\;across\;\text{1}\text{0},\text{0}\text{0}\text{0}\;simulations}{Mean\;of\;\text{1}\text{0},\text{0}\text{0}\text{0}\;simulations}\right)*\text{1}\text{0}\text{0} \end{array}$$

**Fig 5:**
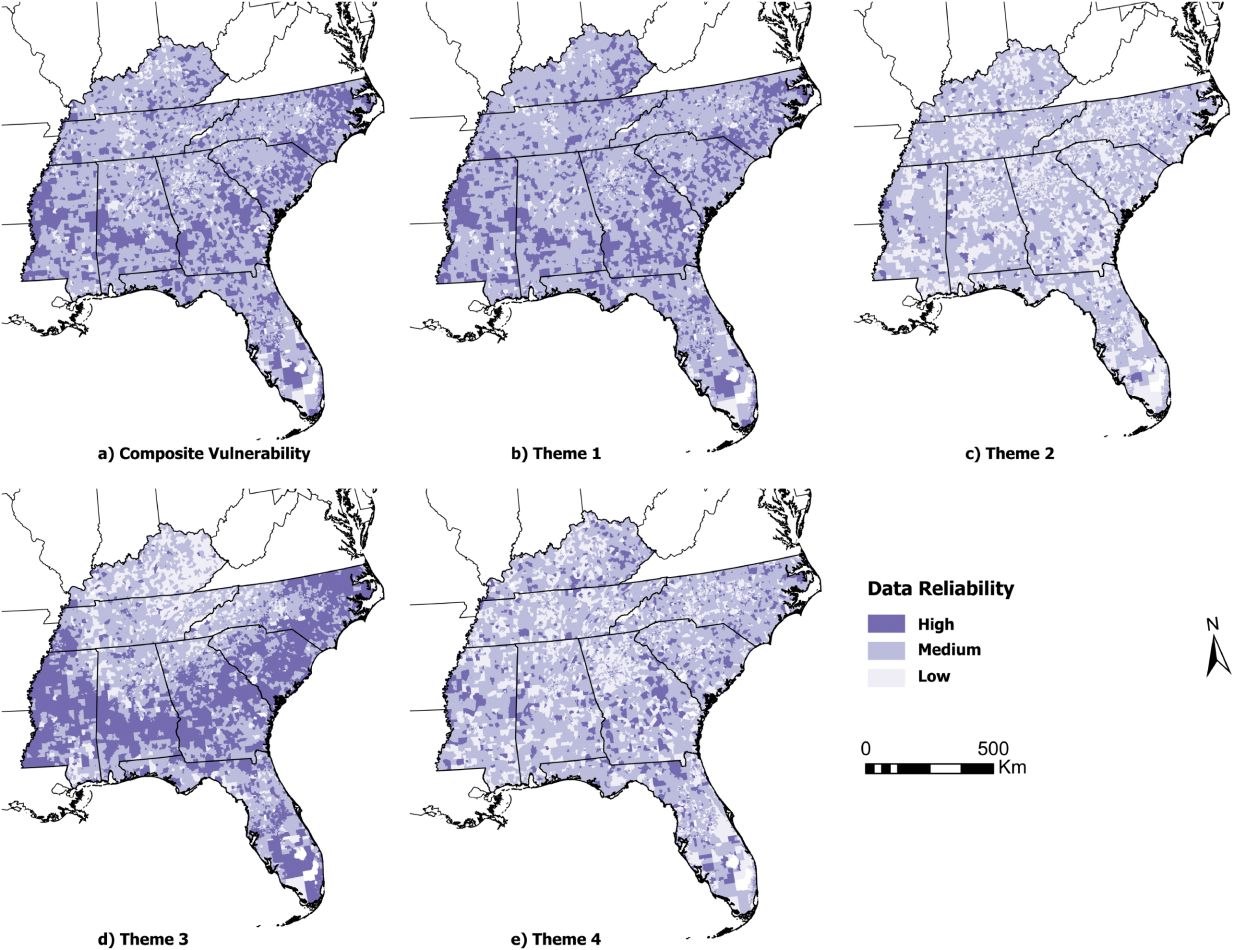
Data reliability according to the CV based Esri standard. Data reliability of a) composite vulnerability, b) Socioeconomic Status, c) Household Characteristics, d) Racial and Ethnic minority Status, and e) Housing Type and Transportation. Geographic boundary files are from IPUMS NHGIS, University of Minnesota. NHGIS citation and use terms were followed. No proprietary basemap tiles, satellite imagery, aerial imagery, or previously published copyrighted map images were used. The mapped classifications and probabilities are author-generated outputs from the SVI-MC analysis.

For the ***composite vulnerability measure***, the overall reliability is good, with most areas showing high or medium reliability (Fig 5a). A few regions, particularly around Atlanta, Birmingham, Huntsville, Nashville, and Raleigh, exhibit low reliability. High reliability is concentrated in the Cotton Belt, in portions of the Carolinas, Georgia, Alabama, and Mississippi. Other scattered areas of low reliability are located in portions of Florida and in scattered pockets in Tennessee and Kentucky. ***Theme 1*** follows a similar pattern to the overall composite vulnerability, with small patches of low reliability scattered across the study area, but most regions show medium to high reliability (Fig 5b). ***Theme 2***, however, displays a different pattern, with low data reliability more prevalent across the study area and very few places with high reliability (Fig 5c). For ***Theme 3***, high data reliability is much widespread, especially in the Cotton Belt. However, large portions of eastern Kentucky and Tennessee, as well as western North Carolina and far northern Georgia, exhibit low reliability (Fig 5d). Finally, ***Theme 4*** presents a mix of low, medium, and high reliability, but a significant portion of the study area has low reliability, especially in the greater Atlanta area. Overall, the data reliability for Theme 4 is more predominant in the medium category (Fig 5e).

We investigated the correlation between the Coefficient of Variation (CV) and the mean composite Social Vulnerability Index (SVI-MC) in the probabilistic model and found a strong negative correlation. The correlation values for overall vulnerability and the four themes are as follows: ***overall vulnerability*** (−0.865), ***theme 1*** (−0.859), ***theme 2*** (−0.889), ***theme 3*** (−0.819), and ***theme 4*** (−0.887). Specifically, as the mean SVI values (or mean theme values) increase, the CV values decrease. Thus, more reliable and less variable SVI values tend to be associated with higher vulnerability index scores.

### Analysis of uncertainty by SVI themes

Hypothesis 1 (H1) stated that the most uncertain and unstable portion of the CDC SVI will be Theme 1, Socioeconomic Status. We conducted formal tests to explore H1 by computing mean CV values for each theme, and we used Welch’s Analysis of Variance (ANOVA) and post-hoc pairwise comparison tests to establish whether Theme 1 was the least reliable and most uncertain amongst the four themes. We used Welch’s ANOVA since it is robust to deviations from normality, especially with a large sample size, and because the test is appropriate when variances are unequal between groups. We also used the Games-Howell Test for post-hoc pairwise comparisons, which is appropriate when data do not meet the assumption of homogeneity of variances, which we confirmed to be the case with Levene’s tests.

The mean CV values were significantly different across themes according to the ANOVA test (F = 4644.3, p < 0.001). Furthermore, all pairwise comparisons provided evidence of statistically significant differences between themes (all with p < 0.001). Theme 2 had the highest mean CV at 37.6 (Table 2), followed by Theme 4 (34.5) and Theme 1 (22.6); Theme 3 (17.2) was lowest. We also plotted the CV values according to Esri’s three-tiered reliability guidance (Table 2). The results indicated that data reliability at the tract level was high (49%) or medium (42%) for Theme 3, leaving only 9% of tracts in the low reliability range. Theme 1 data were also of generally acceptable reliability using CV values, with 27% of tracts in the high and 61% in the medium categories, leaving only 12% in the low reliability category. Themes 2 and 4 had much higher percentages of tracts in the low reliability category, with 39% for Theme 2 and 32% for Theme 4.

**Table 2:** Number (and percentage) of census tracts according to data reliability, as measured using coefficient of variation (CV), within each theme and composite vulnerability.

Census Tracts (n = 17,113)	Composite vulnerability	Theme 1 (mean = 22.6)	Theme 2 (mean = 37.6)	Theme 3 (mean = 17.2)	Theme 4 (mean = 34.5)
High (CV ≤ 12)	5,286 (31%)	4,637 (27%)	1,233 (7%)	8,403 (49%)	2,464 (14%)
Medium (CV 12–40)	9,378 (55%)	10,346 (61%)	9,205 (54%)	7,215 (42%)	9,146 (53%)
Low (CV ≥ 40)	2,449 (14%)	2,130 (12%)	6,675 (39%)	1495 (9%)	5,503 (32%)

These results using CV as our uncertainty measure do not lend support to H1 that Theme 1 would be the least reliable and certain portion of the SVI-MC when accounting for margins of error. Rather, they suggest Themes 2 and 4, respectively, had the highest levels of uncertainty due to sampling error. Theme 3 appears to be the most stable and reliable in this regard, at least for our study area in the southeastern US. The overall compositive vulnerability was relatively similar to the reliability and uncertainty levels evident for Theme 1, with 31% in the high reliability range, 55% in the medium range, and only 14% in the low reliability range (Table 2).

### Entropy as a complementary uncertainty measure

The second hypothesis (H2) stated that information entropy would provide unique uncertainty information in addition to the CV for the purposes of data classification used for mapping. These entropy calculations assume a quintile data classification, which uses ranking of observational units, and then bins each tract into one of five categories for SVI of the MC simulations. Thus, the CV represents variability in SVI measures due to sampling error in the ACS data, and the entropy measure represents variability in ranking and binning within the study area, which varies due to overlapping margins of error in the underlying ACS data. To explore H2, we examined the Pearson correlation coefficient between the CV and entropy for composite vulnerability and the four themes. For the composite SVI-MC scores, there was a significant (α = 0.01), yet weak negative linear relationship (ρ = −0.156) between CV and entropy. Theme 1 showed the same result as for the composite, and Theme 4 was also similar (ρ = −0.119; α = 0.01). Themes 2 (ρ = −0.051) and 3 (ρ = 0.037) both displayed very weak linear relationships between CV and entropy. Therefore, the composite SVI-MC and Themes 1 and 4 demonstrated a slight tendency for greater CVs (more uncertainty due to sampling error) to be associated with lower entropy values (less uncertainty due to variability in quintile binning). However, the two uncertainty measures largely represent unique information, as hypothesized.

To test hypothesis 3, we performed a comparison of means tests to determine if the mean entropy values for composite vulnerability and each of the individual themes in rural and urban tracts were significantly different (Table 3). From the decennial census 2020, we used housing unit data from census table H2 to distinguish between the urban and rural tracts. Tracts having a greater percentage of urban housing than rural housing are considered as urban tracts, and vice versa for designation of rural tracts.

**Table 3:** The mean entropy values for a quantile data classification and the results of Welch’s t-tests comparing the means of rural versus urban census tracts.

	Mean Entropy			
	Rural (n = 4,841)	Urban (n = 12,305)	Welch’s t-test values	P-values
Composite Vulnerability	0.423	0.331	30.319	< 0.001
Theme 1	0.439	0.349	29.056	< 0.001
Theme 2	0.696	0.598	25.429	< 0.001
Theme 3	0.232	0.253	−6.2149	< 0.001
Theme 4	0.608	0.517	24.426	< 0.001

**For *Composite Vulnerability,*** a comparative investigation of normalized quintile entropy between rural and urban areas showed significant results. Rural tracts demonstrated a mean entropy of 0.423, whereas urban tracts had a lower mean entropy of 0.331. This indicates that rural tracts in the southeastern US exhibit less stability in map classifications for the composite SVI, while urban areas demonstrate better stability. In ***Theme 1***, rural areas had a mean normalized entropy of 0.439, while urban tracts exhibited a lower mean entropy of 0.349, indicating increased stability in Theme 1 data classifications for mapping. The statistical significance of the results was confirmed using Welch’s t-test.

***Theme 2*** displayed the highest entropy values averaged across the study area, signifying the least stability in quintile binning of vulnerability index values for the purposes of ranking and/or mapping. Rural regions demonstrated a mean entropy of 0.696, whereas urban regions exhibited a lower mean entropy of 0.598 The Welch’s t-test results provide evidence that rural areas have higher mean entropy than urban areas for Theme 2, indicating greater stability of urban tracts’ vulnerability scores for map classification. For ***Theme 3***, rural tracts had a mean entropy of 0.232, whereas urban tracts had a mean entropy of 0.253. The results of a Welch t-test indicated that urban tracts exhibited a higher mean entropy, signifying less stability in their quintile map classification relative to rural tracts for this theme. Theme 3 was the only instance in which rural tracts had lower entropy than urban tracts.

For ***Theme 4***, rural areas had a mean entropy of 0.608, whereas urban tracts displayed a mean entropy of 0.517. A Welch’s t-test indicated that the average entropy of rural tracts was higher compared to urban tracts, implying that rural tracts demonstrated less stability in quintile data classification for mapping purposes. In summary, rural tracts exhibited higher mean entropy values, signifying greater instability, whereas urban tracts demonstrated lower entropy values. The lone exception was in Theme 3, in which rural tracts had a lower mean entropy compared to the mean of the urban tracts.

To further explore H3, which posited that census tracts in rural areas with smaller sample sizes will be more uncertain and unstable compared to tracts in urban areas, we also conducted spatial analyses of entropy. This decision was warranted because of the spatially smaller census tracts in urban areas, which made it challenging to identify clear patterns in a simple choropleth map. The spatial autocorrelation and clustering results highlighted broader patterns of entropy values across the study region. For this analysis, we utilized the Getis-Ord Gi* statistic and Local Moran’s I, as operationalized in ArcGIS Pro. The two analyses provided complementary results, as the Getis-Ord Gi* analysis identified clusters of higher and lower entropy values, while the Local Moran’s I analysis identified areas of both positive (clusters) and negative (outliers) spatial autocorrelation in entropy values.

For the hotspot analysis (Getis-Ord Gi*), we used a fixed distance band, Euclidean distance, and applied a false discovery rate (FDR) correction. The initial distance band was left empty, and using the geoprocessing tool in ArcGIS Pro, we determined that 50 km was a near-optimal distance threshold, ensuring that each cell had at least one neighbor. We experimented with other distance settings and found that beyond 100 km, the clustering patterns became less interpretable. The most significant hotspots and cold spots, with 99% confidence intervals (CIs), were observed within a 50 km distance bandwidth. For ***Local Moran’s I***, we used similar input parameters as for the hotspot analysis, with the addition of setting the number of permutations. After testing various permutations, we found that 999 permutations provided the most well-defined clusters.

**Fig 6:**
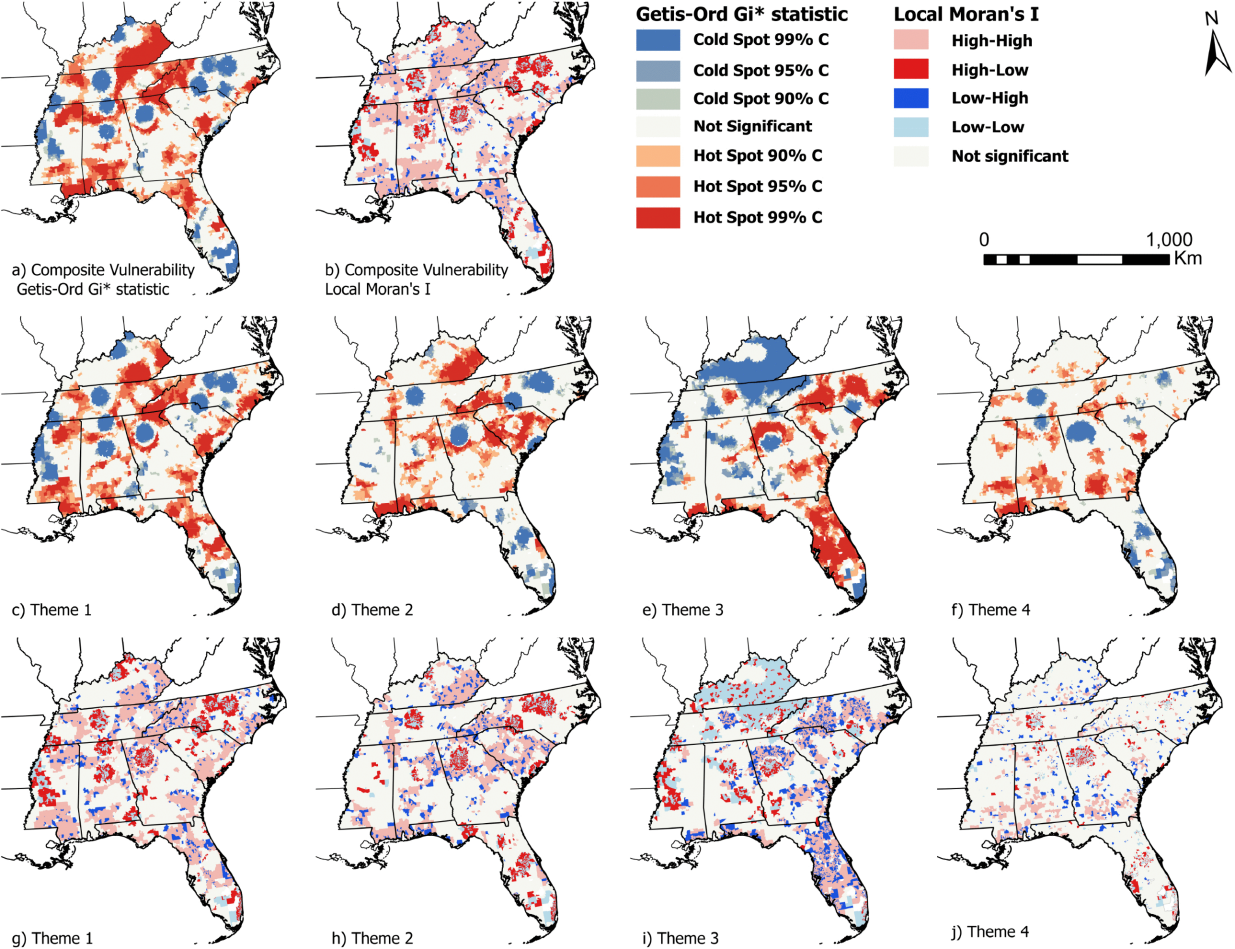
Spatial autocorrelation and clustering based on entropy. Getis-Ord-Gi* statistic (a, c-f) and Local Moran’s I (b, g-j) results for the entropy measure. Geographic boundary files are from IPUMS NHGIS, University of Minnesota. NHGIS citation and use terms were followed. No proprietary basemap tiles, satellite imagery, aerial imagery, or previously published copyrighted map images were used. The mapped classifications and probabilities are author-generated outputs from the SVI-MC analysis.

For the ***Composite Vulnerability (SVI-MC)*** entropy values**,** comparing the Getis-Ord Gi* and Local Moran’s I results, both methods produced similar spatial patterns (Fig 6). The Getis-Ord Gi* map (Fig 6a) showed that most cold spots (99% CI) are in larger metropolitan areas. For example, cold spots are observed in Louisville, parts of greater Cincinnati (in northern Kentucky), Nashville and Memphis (in Tennessee), Huntsville and Birmingham (in Alabama), Atlanta (in Georgia), Charleston (in South Carolina), and Charlotte, Greensboro, and Raleigh-Durham (in North Carolina). Additional cold spots (99% CI) were found in Mississippi’s Delta region, as well as near Jackson (90% CI). In Florida, cold spots were located near Tampa, St. Petersburg, West Palm Beach, Fort Lauderdale, and Miami.

Hot spots, representing higher entropy values and less stability for tracts’ rankings and a quintile map classification, were in areas with lower population densities. For instance, a large swath of hot spots stretched from near Oxford and Holly Springs, Mississippi, into rural west central Tennessee. Another large swath of hot spots was near the Cumberland Plateau in Tennessee, northward to the Daniel Boone National Forest and throughout eastern Kentucky. Additionally, a large hotspot was situated from northeastern Georgia northeastward through the Blue Ridge Mountains of North Carolina. Some hot spots were in hazard-prone near coastal regions, such as Jacksonville, North Carolina, the coastal plains of South Carolina, and Gulf Coast cities like Pensacola, Florida, Mobile, Alabama, and Gulfport-Biloxi, Mississippi. There were also hot spots in Palm Bay, Florida, and areas near Gainesville and Lake City, Florida.

**Fig 7:**
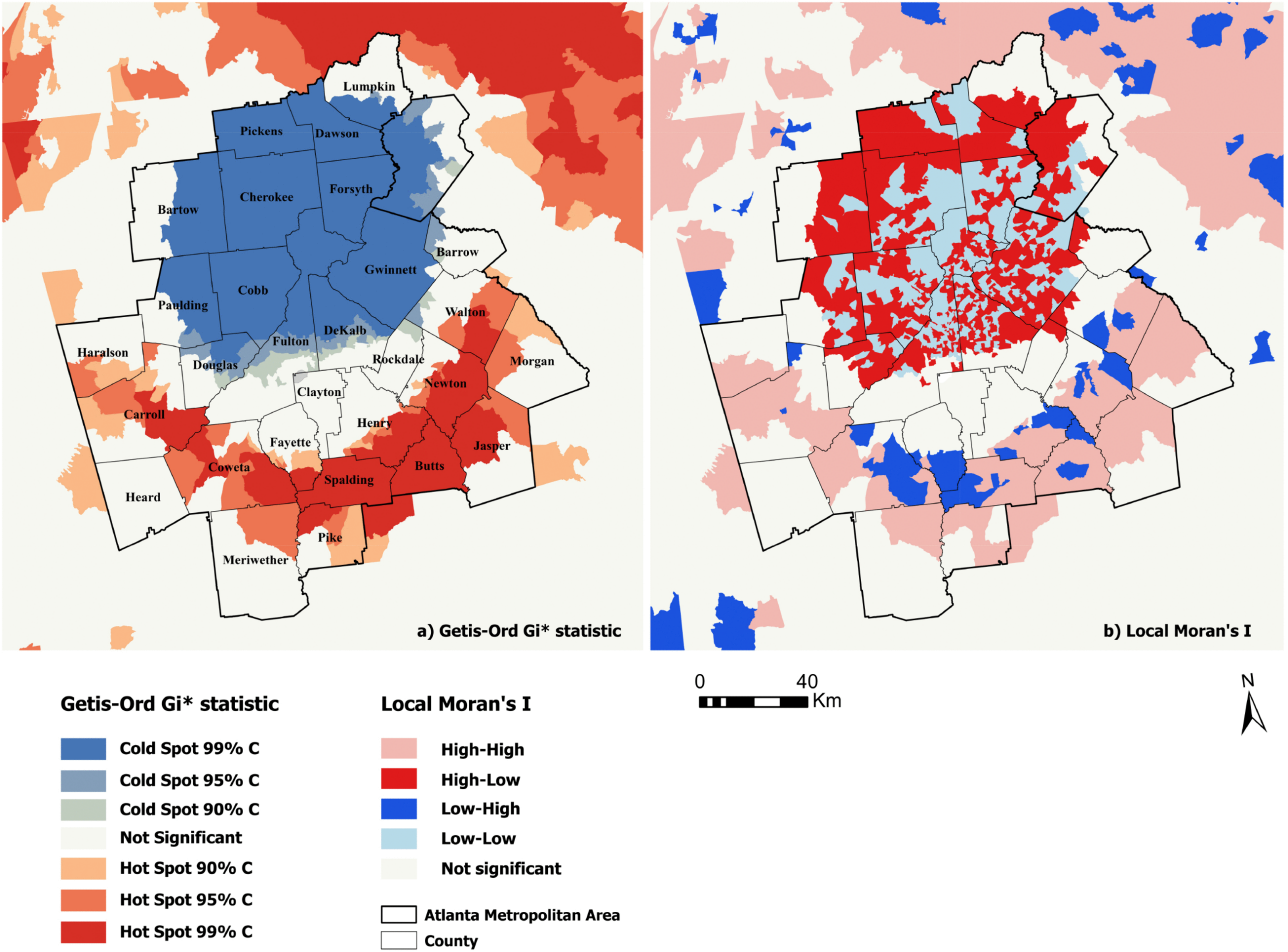
Spatial autocorrelation and clustering for the Atlanta Metropolitan Statistical Area. a) Getis-Ord-Gi* statistic and b) Local Moran’s I entropy results for Composite Vulnerability, zoomed in to the Atlanta, GA Metropolitan Statistical Area. Geographic boundary files are from IPUMS NHGIS, University of Minnesota. NHGIS citation and use terms were followed. No proprietary basemap tiles, satellite imagery, aerial imagery, or previously published copyrighted map images were used. The mapped classifications and probabilities are author-generated outputs from the SVI-MC analysis.

Local Moran’s I Anselin cluster analysis (Fig 6b) revealed a similar pattern of high-high and low-low clusters as seen in the Getis-Ord Gi* results. For example, high-high clusters were found around the Appalachian Mountains, Blue Ridge Mountains, Cumberland Plateau, and several locations along the Gulf Coast and Atlantic Coast. Low-low clusters are concentrated in major cities such as Atlanta, Birmingham, Huntsville, Nashville, Louisville, Charlotte, Raleigh, and Miami. Surrounding these cities were high-low clusters, indicating that while these metropolitan areas have low entropy (more stable data and map classification), the outskirts of these cities showed higher entropy (less stable). This suggests that while core regions of larger metropolitan areas were well-represented in the ACS data, the outlying suburban and exurban areas surrounding them may not have been. For example, zooming in to the Atlanta, GA area, census tracts with greater entropy (less stability) were concentrated to the north of the Atlanta Metropolitan Statistical Area (MSA) county boundaries and in an arc across the southern one-third of the Atlanta MSA (Fig 7). Tracts with lower entropy values (more stability) were clustered mainly in the northern half of the Atlanta MSA (Fig 7a), though there are high-low outliers near the core low-low entropy clusters, especially in northern Fulton, DeKalb, Gwinnett, Forsyth, Cherokee, and Cobb counties (Fig 7b).

***Theme 1*** is focused on socioeconomic status indicators. Cold spots were concentrated in large cities, like the composite vulnerability entropy pattern (Fig 6c). This makes sense because these variables are typically concentrated in urban areas. Poverty, housing cost burdens, and lack of education are common in cities, and people often forgo health insurance due to low incomes and high living expenses. Therefore, the data for these variables was well-represented in larger cities. Hotspots, conversely, were in sparsely populated rural areas and hazard-prone coastal regions. Local Moran’s I showed a similar pattern (Fig 6g), with low-low clusters in several urban cores and high-low clusters surrounding them. High-high clusters were found in mountainous and coastal areas, while low-high clusters were widely scattered across the study area.

***Theme 2*** represented household vulnerability characteristics such as persons aged 65 and older, persons aged 17 and younger, the disabled, single-parent households, and individuals with limited English proficiency. In this theme, we observed fewer distinct hot spots and cold spots (Fig. 6d) compared to Theme 1. Significant (99% CI) cold spots were located in Atlanta, Nashville, Charlotte, Raleigh, and Orlando, Florida. Large parts of the study area show no significant clustering. Hot spots were concentrated in eastern Kentucky, western North Carolina, much of South Carolina and the Augusta, Georgia area, as well as coastal Mississippi and Alabama. Local Moran’s I showed a similar pattern (Fig 6h), with low-low clusters in major cities and high-high clusters mainly in mountainous and coastal areas.

***Theme 3*** consisted of a single variable that encompasses multiple race and ethnicity categories, including Hispanic or Latino, Black or African American, American Indian and Alaska Native, among others. Most cold spots with low entropy values were in the mostly rural portions of Kentucky and Tennessee (Fig 6e), where the population is predominantly white. Other cold spots were near and south of Atlanta, near Memphis, the Mississippi Delta region, southern Alabama, and near Miami, all areas with significant Black and African American populations. Many Florida tracts were classified as hot spots, with low-low clusters around Miami and high-high clusters and low-high outliers spread throughout the rest of the state (Fig 6i). The Moran’s I result also showed notable high-low outlier tracts spread across the medium-sized towns and cities of Kentucky and eastern Tennessee.

***Theme 4*** included housing type and transportation indicators such as housing in structures with 10 or more units, mobile homes, overcrowded housing, households without vehicles, and group quarters. Less of the study area was statistically significant in spatial analyses for this theme, yet distinct cold spots were present near Atlanta, Nashville, Huntsville (Alabama), Miami, Tampa, and Orlando (Fig 6f). Some regional entropy hot spots were in southern Mississippi, Alabama, Georgia, and South Carolina. The Local Moran’s I map (Fig 6j) indicated significant low-low clusters in central Atlanta and Nashville, surrounded by high-low outliers. High-high clusters and low-high outliers were scattered across the study area mostly clearly in a swath from southern Georgia to southern Mississippi.

### Probabilistic approach to identification of the most and least vulnerable tracts

Our probabilistic SVI-MC model, developed using 10,000 simulations, allowed additional exploration of the upper and lower extremes of the vulnerability index. As part of our entropy calculations, we established the percentages for census tracts to fall within specific vulnerability quantiles across the 10,000 simulations. The entropy values were calculated using quintiles that were defined according to the percentile rank values, with five categories: very low (0.0–0.2); low (0.2–0.4); medium (0.4–0.6); high (0.6–0.8); and very high (0.8–1). Additionally, because the CDC flags tracts for special attention if they have an SVI value between 0.9 and 1, we also focused on this 90^th^ percentile threshold, as well as the 10^th^ percentile threshold on the lowest vulnerability end of the SVI spectrum.

In comparing our probabilistic results from SVI-MC against the deterministic values from the CDC SVI, there was relatively good agreement about which tracts would be considered the highest priority as the topmost 10% or 20% socially vulnerable tracts in the study area. For the Very High quintile, there was about a 68% correspondence between the tracts in the CDC SVI upper quintile and tracts which had their maximum quintile probability as the upper quintile in SVI-MC (Table 4). The overlap percentage between the two models was almost identical (68%) for the lower quintile, those tracts that would be considered the least socially vulnerable. However, these results also mean that roughly one-third of tracts considered to be among the most (or least) vulnerable in the CDC SVI, were more likely to be shuffled into the middle of the distribution according to the SVI-MC quintile probabilities. Even more exclusive to the high end of the CDC SVI, the correspondence was slightly less at about 58%, thus leaving about 40% of tracts that were less likely to fall in ^th^e 90^th^ percentile in SVI-MC. The three middle quintiles had overlap percentages closer to 40%. Not surprisingly, these middle quintiles saw much more shuffling of tracts in the 10,000 simulations than at the lower and upper ends of the SVI scores (Table 4).

**Table 4:** Number of census tracts falling into five vulnerability quintiles, as well as the 90^th^ percentile, according to data from the CDC SVI and SVI-MC models.

	CDC SVI	SVI-MC Maximum Probability	Consistency of results across both models
Very low (0.0–0.2)	3,414	3,295	2,326 (68.1% of CDC SVI)
Low (0.2–0.4)	3,416	3,432	1,397 (40.9% of CDC SVI)
Medium (0.4–0.6)	3,416	3,460	1,298 (38.0% of CDC SVI)
High (0.6–0.8)	3,417	3,618	1,474 (43.1% of CDC SVI)
Very high (0.8–1.0)	3,418	3,335	2,337 (68.4% of CDC SVI)
90^th^ percentile (0.9–1.0)	1,709	1,579	986 (57.7% of CDC SVI)

**Fig 8:**
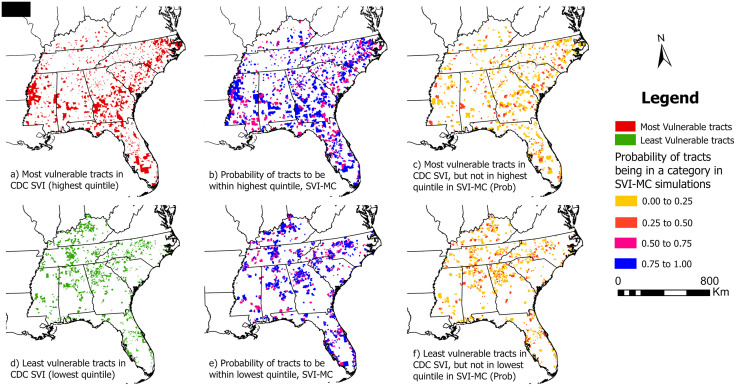
Probability of tracts being in the vulnerability quintile in SVI-MC. Geographic boundary files are from IPUMS NHGIS, University of Minnesota. NHGIS citation and use terms were followed. No proprietary basemap tiles, satellite imagery, aerial imagery, or previously published copyrighted map images were used. The mapped classifications and probabilities are author-generated outputs from the SVI-MC analysis.

We further explored the probabilistic results via mapping, with focus on the most and least vulnerable census tracts in the study area, defined as the very low (0.0–0.2) and very high (0.8–1.0) quintiles. The spatial patterns of the most vulnerable tracts (very high), according to the deterministic results from the CDC SVI (Fig 8a), resembled the results from our probabilistic SVI-MC model (Fig 8b). The SVI-MC results for the very high quintile suggested more prevalence of higher social vulnerability near Atlanta, GA, the Piedmont of the Carolinas, eastern Tennessee and Kentucky, and in portions of central Florida, in comparison to the CDC SVI results. Tracts that were in the very high quintile in CDC SVI, but not likely to be in the very high quintile in SVI-MC, were widely scattered across the study area (Fig 8c). Perhaps portions of the coastal plain of Georgia and the Carolinas, as well as portions of northeastern Mississippi, were overestimated in the CDC SVI map; however, there was not a clear spatial pattern that suggested without doubt that the CDC SVI overestimated the social vulnerability of a specific region.

The spatial patterns of the least vulnerable tracts (very low), according to the deterministic CDC SVI model (Fig 8d), were much like the results from the SVI-MC model (Fig 8e). The SVI-MC results for the very low quintile suggested more prevalence of lower social vulnerability tracts in southern Florida and coastal areas of Georgia and South Carolina, in comparison to the CDC SVI results. Tracts that were in the very low quintile in CDC SVI, but not likely to be in the very low quintile in SVI-MC, were prominent in northern Alabama and Georgia, as well as near Charlotte and Raleigh, North Carolina (Fig 8f). Other cities where social vulnerability was likely underestimated when comparing the CDC SVI map and the SVI-MC maps included Knoxville and Nashville, Tennessee, Lexington, Kentucky, and Orlando and Jacksonville, Florida. Another interesting finding is that some tracts (shaded in orange) had very low probabilities (< 25%, some even near 0%) of falling within the highest quintile (Fig 8c) or within the lowest quintile (Fig 8f) in the SVI-MC simulations even though they fell within one of those extreme categories according to CDC SVI.

## Discussion

This study advances the quantification of uncertainty in social vulnerability indices through two main contributions. First, we quantified the uncertainty in a widely used social vulnerability index that results from sampling error in the American Community Survey (ACS) data and examined how this uncertainty influences interpretation and communication of index values. We introduced a probabilistic extension of the CDC’s Social Vulnerability Index (SVI), the SVI-MC model, using 10,000 Monte Carlo simulations. This approach embeds the ACS margins of error into index computation, producing uncertainty-aware vulnerability estimates across eight southeastern U.S. states and more than 17,000 census tracts. Rather than replacing the CDC SVI or producing substantially different mean scores, the SVI-MC framework preserves the underlying structure of the CDC index while enabling probabilistic evaluation of uncertainty around its scores, rankings, and classifications.

Our approach complements recent work that has begun to foreground uncertainty propagation in vulnerability and composite-index construction. Feldmeyer et al. demonstrated that sampling error can propagate through composite indicators and interact with methodological choices such as normalization, aggregation, and weighting [40]. Arabadjis et al. similarly showed that uncertainty can destabilize interpretation in a hazard-specific wildfire risk index [39]. However, their social vulnerability component was based on a single ACS-derived indicator, population aged 65 and older, combined with a remotely sensed locational vulnerability indicator. Our study extends this emerging literature by applying uncertainty propagation to the full CDC SVI framework, including all 16 ACS-derived indicators, four thematic subindices, percentile rank transformations, final composite rankings, and mapped classifications. This distinction is important because the CDC SVI is widely used as an operational tool in emergency management, public health, and disaster planning, where small changes in ranking or classification can affect how vulnerability is interpreted, communicated, and visualized.

Second, we demonstrated that information entropy complements traditional measures of uncertainty such as the coefficient of variation (CV) by capturing a distinct dimension of uncertainty: instability in ranking and categorical classification caused by overlapping margins of error. While the CV summarizes the magnitude of variability, entropy reveals how frequently spatial units shift among categories in a ranked or binned framework, such as quintiles commonly used in choropleth mapping. This dual-metric approach provides a fuller representation of uncertainty than approaches relying only on dispersion metrics. It also has practical value for map interpretation and decision-making in resource allocation, emergency management, and public health, where understanding the stability of category assignments is crucial.

Our findings also reveal several substantive insights. Contrary to our expectation (H1), Theme 1 (Socioeconomic Status) was not the most uncertain; Themes 2 (Household Characteristics) and 4 (Housing and Transportation) exhibited higher uncertainty due to sampling errors. This aligns partially with findings from previous research [44] that found heterogeneity across socioeconomic variables but also extends that insight by identifying where and why classification instability occurs spatially. These results suggest that researchers and practitioners might consider weighting or down-weighting indicators according to data reliability when constructing composite indices.

Comparisons of urban and rural entropy further emphasize the spatial nature of uncertainty: rural tracts exhibited greater instability in vulnerability rankings, consistent with prior work noting larger ACS margins of error in sparsely populated areas [34]. The geospatial clustering analyses (Getis-Ord Gi* and Local Moran’s I) revealed systematic hot spots of high entropy in rural, mountainous, and coastal regions, indicating that vulnerability estimates in such locations should be interpreted cautiously. Urban cores were more stable, whereas surrounding suburban and exurban tracts often showed higher entropy. Further research could explore other classification methods (e.g., natural breaks or others) to reduce instability in rankings and categories based on quantile classification.

Finally, direct comparison between the CDC SVI and our probabilistic SVI-MC revealed that while roughly 68 percent of tracts matched in the highest and lowest vulnerability categories, about one-third shifted ranks across simulations. This partial misalignment underscores a key message: deterministic vulnerability scores can overstate confidence in classification results. Our analysis therefore provides an uncertainty benchmark for future SVI applications and a framework adaptable to other ACS-based composite indices.

Collectively, these contributions establish the novelty of the SVI-MC framework as a replicable, probabilistic, and spatially explicit approach that both quantifies and visualizes uncertainty in social vulnerability modeling. By embedding aleatoric uncertainty into index construction and by coupling variance-based and entropy-based measures, this study advances methodological precision and supports more defensible use of vulnerability indices in research and policy.

## Conclusion

This study advanced the understanding of aleatoric uncertainty in social vulnerability indices by examining how sampling error in ACS input data propagates through the CDC Social Vulnerability Index (SVI). We introduced SVI-MC, a probabilistic approach that integrates the margins of error from ACS data into the CDC SVI framework using Monte Carlo simulations. While SVI-MC (using the mean output) and the CDC SVI yield similar overall patterns, our model allows for deeper insights into uncertainty surrounding index values, rankings, and mapped classifications. Our findings show that Theme 2 (Household Characteristics) exhibits the greatest uncertainty, while Theme 3 (Racial and Ethnic Minority Status) is the most stable. Rural, mountainous, and coastal regions exhibited greater classification instability, whereas urban areas were generally more stable. Additionally, we introduced an entropy-based measure that complements the coefficient of variation (CV) by capturing instability in categorical rankings caused by overlapping margins of error. This provides a more nuanced view of uncertainty, especially for common data classification methods used in mapping social vulnerability.

SVI-MC represents a replicable and adaptable framework for uncertainty-aware social vulnerability assessment. The method has potential applications for other ACS or survey-based composite indicators and is especially relevant for finer geographic scales, where sampling uncertainty is often more pronounced. By integrating probabilistic simulation with multiple uncertainty measures, our approach improves the transparency and interpretability of vulnerability indices and supports more defensible use of these tools in hazard management, emergency response, public health, and resource allocation. Future work should expand this approach nationwide, first at the census tract level and then at finer spatial resolutions, to further evaluate how ACS sampling uncertainty affects social vulnerability modeling across different geographic contexts.
